# SGLT2 inhibitor dapagliflozin reduces proximal tubular cell damage biomarkers in patients with acute heart failure

**DOI:** 10.1080/0886022X.2024.2373275

**Published:** 2024-07-05

**Authors:** Pongsathorn Gojaseni, Jananya Wattanakul, Anan Chuasuwan, Anutra Chittinandana

**Affiliations:** Division of Nephrology, Department of Medicine, Bhumibol Adulyadej Hospital, Directorate of Medical Services, Royal Thai Air Force, Bangkok, Thailand

Acute heart failure (AHF) is characterized by new-onset or worsening symptoms of heart failure, often leading to hospitalization and mortality. Current treatment options for AHF mainly focus on relieving symptoms and reducing fluid overload, which can have potentially harmful effects on kidney function. Consequently, there is a need for the development of novel and inventive therapies aimed at improving both cardiovascular and kidney outcomes in individuals with AHF.

Sodium-glucose co-transporter 2 (SGLT2) inhibitors have emerged as a disease-modifying therapy to reduce cardiovascular events and prevent the progression of chronic kidney disease (CKD) [1]. The rapid growth of clinical trials on SGLT2 inhibitors has expanded the approved clinical indications for these agents. The recent clinical trials [2,3] have shown that SGLT2 inhibitor have reduced the risk of cardiovascular death in the setting of AHF. However, physicians are still hesitant to prescribe SGLT2 inhibitors to AHF patients during their hospitalization primarily due to the transient rise in serum creatinine [4].

Nevertheless, there are limitations associated with employing serum creatinine as a diagnostic tool for detecting AKI in the context of AHF due to its delayed response as a marker of tubular injury. Moreover, increased serum creatinine levels in AHF do not always indicate renal tubular cell injury. As a result, many tubular injury biological markers have been used for the early detection of AKI in this setting. Of these, the use of tissue inhibitors of metalloproteinases-2 [TIMP-2] and insulin-like growth factor-binding protein 7 [IGFBP-7] is one of the most evidence-based [5].

In this study, we conducted a randomized controlled trial to investigate the protective effect of SGLT2 inhibitors on proximal tubular cell damage biomarkers (urinary [TIMP-2] × [IGFBP-7]) in patients hospitalized for AHF. The primary objective of this study was to compare the change of urinary [TIMP-2] × [IGFBP-7] in SGLT2 inhibitor addon to standard of care with standard of care alone. The incidence of AKI, change from baseline serum creatinine, length of stays, cumulative urine output, and adverse events were also assessed.

The enrollment period took place from August 2022 to January 2023. Out of the initial 32 patients who underwent randomization, 7 were subsequently excluded from the study due to incomplete biochemical data in the first week, comprising 5 cases discharged and 2 case died. Consequently, 25 eligible patients were enrolled for analysis (12 dapagliflozin, 13 control). The mean age was 67.1 ± 15.1 years, 48.5% were male, the mean ejection fraction was 42.1 ± 16.5% and the median NT-proBNP was 5,359 pg/ml (interquartile range 3,496 − 10,843). The most common precipitating factors of AHF were salt and water retention (36%), followed by acute coronary syndrome (28%). Other underlying comorbidities and medications at baseline were balanced between treatment groups (Table S1).

The mean ± standard error (SE) of urinary [TIMP-2] × [IGFBP-7] in the dapagliflozin and control group were 0.223 ± 0.165 vs 0.073 ± 0.048 (*p* = .340) at the enrollment. Compared with control group, dapagliflozin group significantly reduced urinary [TIMP-2] × [IGFBP7] after 7 days [dapagliflozin: −0.03 ± 0.11 (ng/mL)^2^/1000; control: +0.4 ± 0.14 (ng/mL)^2^/1000; *p* = .022]. After a 28-day follow-up period, 7 patients (3 in the dapagliflozin, 4 in the control group) were discharged home and 2 patients died (1 from each group), leaving a total of 16 patients with complete data available. The mean change in urinary [TIMP-2] × [IGFBP-7] in dapagliflozin group tended to be decreased compared to controls. [dapagliflozin: −0.09 ± 0.28 (ng/mL)^2^/1000; control: +0.67 ± 0.32 (ng/mL)^2^/1000; *p* = .096] (Figure 1). Furthermore, we conducted additional analysis using a threshold of 0.3 (ng/ml)^2^/1000, a value that showed a strong association with AKI. We observed a trend where the number of patients with urinary [TIMP-2] × [IGFBP-7] levels exceeding 0.3 was slightly lower in the dapagliflozin group, although this difference did not reach statistically significant (25.0% vs 53.8%, *p* = .288) (Table 1).

**Figure 1.**
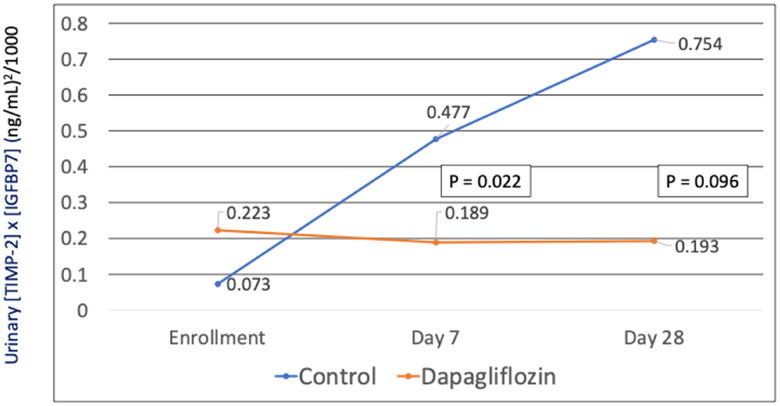
Effects of dapagliflozin on urinary [TIMP-2] × [IGFBP-7] in patients with acute heart failure treated with dapagliflozin (*n* = 12, orange line) or control (*n* = 13, blue line). Data are shown as mean urinary [TIMP-2] × [IGFBP-7] at baseline, after 7, and 28 days. *p*-value compared changes from baseline between both groups.

**Table 1.** Secondary outcomes and adverse events.

Outcomes	Dapagliflozin (*n* = 12)	Control (*n* = 13)	*p*-value
Acute kidney injury (%)	4(33.3)	6(46.2)	.513
AKI by creatinine criteria (%)			
AKI stage I	4(33.3)	6(46.2)	.513
AKI stage II	0	0	–
AKI stage III	0	0	–
AKI by onset (%)			
0–3 days	4(33.3)	2(15.4)	.561
4–7 days	0(0)	2(15.4)	.497
>7 days	0(0)	2(15.4)	.497
Predicted AKI by urinary [TIMP-2] × [IGFBP-7] criteria* (%)	3(25)	7(53.8)	.288
AKI requiring dialysis	0	0	–
Mean urine output (first 3 days), ml, mean ± SE	2,237.0 ± 876.1	1,855.9 ± 473.5	.184
Length of stay, days, median (IQR)	4(3–4)	5(3–11)	.141
28-day mortality (%)	1(8.3)	1(7.7)	1.0
Revisit in 28-day (%)	1(8.3)	1(7.7)	1.0
Adverse events (%)			
Urinary tract infection	1(8.3)	2(15.4)	1.0
Hypokalemia	8(66.7)	8(53.8)	.839
Hypomagnesemia	6(50.0)	4(30.8)	.622
Hypotension	0	1(7.7)	1.0
Hepatitis	2(16.7)	2(15.4)	.656

* Predicted AKI by urinary [TIMP-2] × [IGFBP-7] criteria using a threshold of 0.3 (ng/ml)^2^/100.

Incidence of AKI by serum creatinine from KDIGO criteria in the dapagliflozin and control group was 33.3% and 46.2%, respectively (*p* = .513) (Table 1). It was found that AKI occurrences in this study were generally less severe; all cases were classified as KDIGO stage 1, and none required dialysis. Further analyses showed no difference between the dapagliflozin and control group for the change from baseline serum creatinine, length of stay, and adverse events (Table S1 and Figure S3). The cumulative urine output during the first 3 days was not statistically different between both groups (8,587 ± 1,346 vs 8,060 ± 1,090 mL; *p* = .420) (Figure S4). However, dapagliflozin group demonstrated a trend toward lower cumulative furosemide dose compared with control group. (61.94 ± 10.37 vs 104.62 ± 21.92 mg/day; *p* = .100).

In discussion, this study demonstrated that initiation of dapagliflozin significantly decrease urine [TIMP-2] × [IGFBP7] during the first week, that supported the protective effect of SGLT2 inhibitor on renal tubular injury. The results are consistent with study from Thiele K, et al. that showed the reduction of urine [TIMP-2] × [IGFBP7] after initiation of empagliflozin in AHF patients for 3 days [6]. Thus, we may conclude that SGLT2 inhibitor can improve tubular cell injury in patients hospitalized for acute heart failure since urinary [TIMP-2] × [IGFBP-7] is the biomarker of cell cycle arrest during the early phase of proximal tubular damage.

The proximal tubule accounts for the highest oxygen consumption in the kidney because the substantial energy demand associated with the reabsorption of electrolytes and organic solutes within this tubular segment. Moreover, tubular hypoxia is a crucial factor in the pathophysiology of acute tubular necrosis [7]. It disrupts normal kidney function, triggers cellular damage and inflammation, and ultimately leads to AKI. Administration of SGLT2 inhibitors could abolish this effect. Previous studies demonstrated that SGLT2 inhibition in type 2 diabetes patients improved renal cortical oxygen tension, potentially benefiting the tubular cell integrity [8]. These results were collaborated by meta-analysis, which found that SGLT-2 inhibitor was associated with a reduced risk of AKI in various settings [9].

This study also demonstrated that SGLT2 inhibitor is safe for early administration in patients hospitalized for AHF. Although dapagliflozin initiation in AHF initially leads to a transient increase in serum creatinine, but this effect stabilizes overtime. Furthermore, dapagliflozin has demonstrated the capacity to improve kidney function within the first few weeks in most patients. These findings align with EMPULSE study [3] and the substudy of EMPA-RESPONSE-AHF [4].

In terms of secondary outcomes, both groups did not show a statistically significant in cumulative urine output, but dapagliflozin group demonstrated a tendency for reduced cumulative furosemide dose. The length of stay and adverse events such as urinary tract infection, hepatitis and electrolyte imbalance were not different in both groups. Taking all the data together, we can conclude that SGLT2 inhibitors are safe for early administration in hospitalized patients with AHF.

Limitations of our study include small sample size and use of a surrogate measure for the primary outcome. Moreover, some enrolled patients were lost to follow-up after discharge from the hospital. Thus, the power of study at the end must be a point of concern. Therefore, A larger prospective study should be conducted to investigate the potential benefits of this intervention on clinical outcomes.

In conclusion, initiation of SGLT2 inhibitors in patients with AHF significantly decrease the urinary AKI risk markers TIMP-2 and IGFBP7 within the first week, supporting the protective effect of SGLT2 inhibitor on renal tubular injury. However, it should be noted that during this period, serum creatinine levels may slightly increase. Nevertheless, these findings provide physicians with confidence to prescribe the medication in such settings.

## Supplementary Material

Supplemental Material

## Data Availability

The data underlying this article will be shared on reasonable request to the corresponding author (P.G.).

## Additional information

The manuscript underwent modifications from an earlier version that had been posted on the preprints server (https://www.researchsquare.com/article/rs-3869067/v2) and presented at ASN 2023 in Philadelphia.
