# Supplementary material for: SGLT2 inhibitor dapagliflozin reduces proximal tubular cell damage biomarkers in patients with acute heart failure
Source: Ren Fail. 2024 Jul 5;46(2):2373275. doi: 10.1080/0886022X.2024.2373275 (PMC467088; doi:10.1080/0886022X.2024.2373275)
Supplement: Supplemental Material [file IRNF_A_2373275_SM5609.docx]

**Supplementary Materials**

**SGLT2 Inhibitor Dapagliflozin Reduces Proximal Tubular Cell Damage Biomarkers in Patients with Acute Heart Failure**

Pongsathorn Gojaseni^1^, Jananya Wattanakul^1^, Anan Chuasuwan^1^, Anutra Chittinandana^1^

^1^Division of Nephrology, Department of Medicine, Bhumibol Adulyadej Hospital, Directorate of Medical Services, Royal Thai Air Force

**Supplementary Methods**

**Study design**

We conducted a randomized prospective open-label controlled trial of patients hospitalized for AHF in Bhumibol Adulyadej Hospital, Directorate of Medical Services, Royal Thai Air Force. The study protocol was approved by the Institutional Review Board of Bhumibol Adulyadej Hospital with approval number 46/65 and was registered in the Thai Clinical Trials Registry (TCTR) with registration number TCTR20221003002. This study was conducted in accordance with the ethical principles of the 1964 Declaration of Helsinki and its later amendments. The investigators informed patients or their surrogates concerning the study orally and written informed consent was given before entry into the study.

Inclusion criteria were aged ≥ 18 years, presence of AHF requiring hospital admission as well as serum level of N-terminal pro-brain natriuretic peptide (NT-proBNP) ≥ 1600 pg/ml

or ≥ 2000 pg/ml in patients with atrial fibrillation regardless of ejection fraction or diabetes status. Key exclusion criteria were: patients with type 1 diabetes, history of diabetic ketoacidosis or ketosis, estimated glomerular filtration rate by the CKD Epidemiology Collaboration [eGFR (CKD-EPI)] < 30 ml/min/1.73m^2^, patients with cardiogenic shock, septic shock or systolic blood pressure < 100 mmHg, patients with intravenous vasopressor, vasodilators or inotropic drugs within 6 hours, planned for percutaneous coronary intervention therapy, acidemia (blood pH < 7.2), and liver failure. In terms of calculating the sample size, the following assumptions were considered: power 0.8, alpha 0.05, and standard deviation of urinary [TIMP-2] x [IGFBP-7] of 0.47 (ng/ml)^2^/1000. From these assumptions, a total sample size of 22 would enable the detection of a 0.6 (ng/ml)^2^/1000 difference in the change from baseline of urinary [TIMP-2] x [IGFBP-7] based on Thiele K, et al. [S1].

**Primary and secondary outcomes**

The primary outcome of this study is the change from baseline of urinary [TIMP-2] x [IGFBP-7]. Statistical analysis was based on detecting a difference in urinary [TIMP-2] x [IGFBP-7] change from baseline between the dapagliflozin group and controls. The secondary outcome is the incidence of AKI by KDIGO creatinine criteria, the change from baseline creatinine, length of stays, cumulative urine output, and adverse events.

**Trial procedures**

Patients were randomized into 2 groups using a random block of 4 allocation method. The dapagliflozin group received dapagliflozin 10 mg on top of standard of care within 24 hours after enrollment, while the control group received standard of care alone. Baseline information including demographic data, comorbidities and current medication were recorded. Laboratory investigations were performed at baseline and 1 day, 2 days, 3 days, 7 days, 14 days, and 28 days following treatment initiation as shown in **Figure S1**. The urine sample was collected then centrifuged and frozen at -80 °C and then urinary [TIMP-2] x [IGFBP-7] was performed by NephroCheck^®^ using the VITROS 5600 Integrated System (Astute Medical, San Diego, CA, USA) at baseline, on day 7, and on day 28. The outcome including length of stay, cumulative urine output, adverse events including the gastrointestinal system, urinary tract infection, electrolyte imbalance, discontinuation of treatment and date of death were collected. All patients received treatment in accordance with the ESC guidelines for the diagnosis and treatment of acute heart failure [S2].

**Relevant Definition**

Kidney disease improving global outcome (KDIGO) serum creatinine criteria were used to classify AKI status [S3]. The baseline serum creatinine was the first creatinine recorded on the day of enrollment. AHF refers to heart failure that occurs suddenly or a stable condition that then becomes worse within a short period of time, according to the criteria of the European Society of Cardiology guidelines (ESC) [S2].

**Statistical Analysis**

The study used IBM SPSS Statistics Version 22 (Chicago, IL, USA) program for data analysis and data processing. The categorical data were reported as the number with a percentage. The continuous data were reported as mean ± standard deviation (SD), mean ± standard error (SE), or median (interquartile range, IQR) as appropriate. Fisher’s exact test or Chi-square test were used for categorical variable and student’s *t-test* or Mann Whitney U test for continuous variable. We used a P value < 0.05 as statistically significant.

**Supplementary Figures**

**
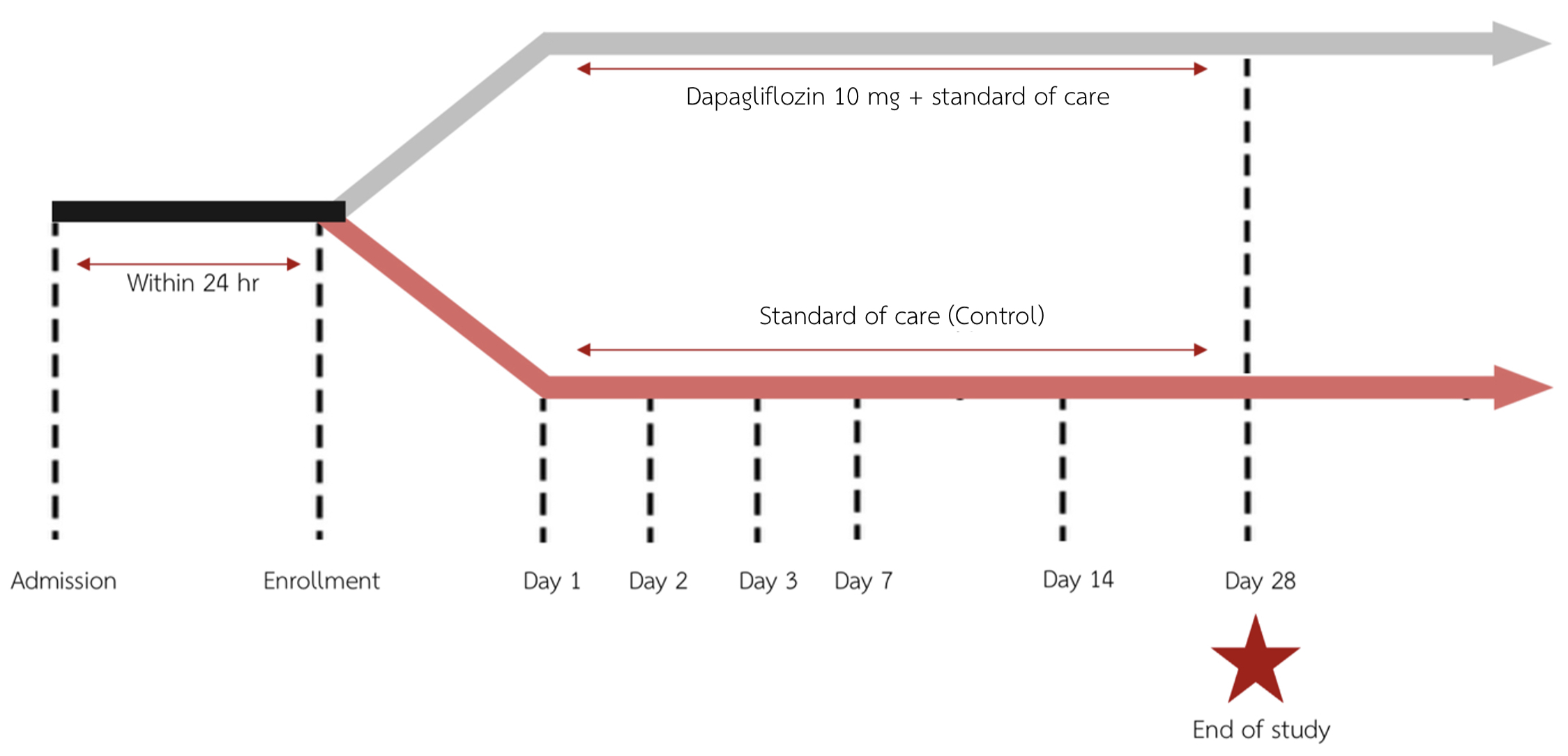
**

**Figure S1**: Protocol and timeline of the study


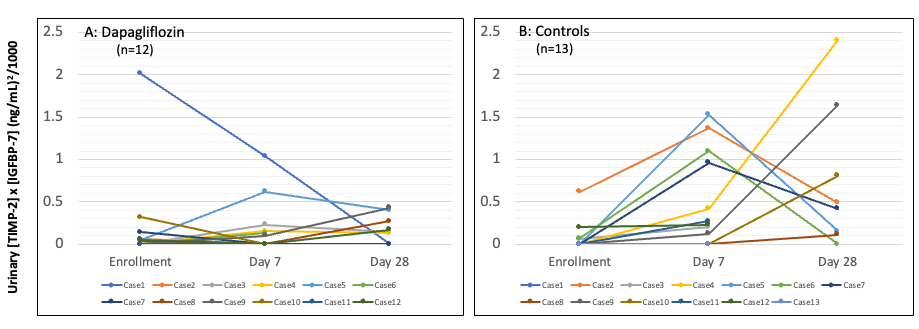


**Figure S2**: The changes in urinary [TIMP-2] x [IGFBP-7] at baseline, after 7, and 28 days in each patient; A: dapagliflozin group (n = 12); B: control (n = 13).

**
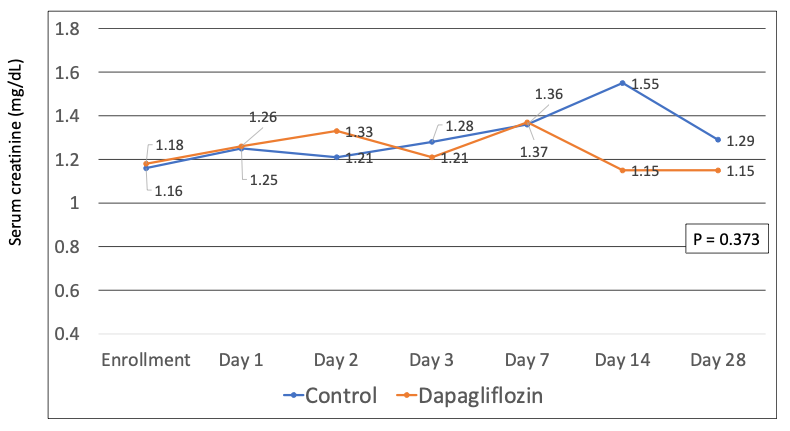
**

**Figure S3**: Effects of dapagliflozin on serum creatinine in patients with acute heart failure treated with dapagliflozin (n = 12, orange line) or control (n = 13, blue line). Data are shown as mean serum creatinine at baseline, after 1, 2, 3, 7, 14, and 28 days. p-value compared changes from baseline between both groups.

**Figure S4**: The mean of cumulative urine output during the first 3 days in patients with acute heart failure treated with dapagliflozin (n = 12, orange line) or control (n = 13, blue line). p-value compared the cumulative urine output at day 3.

**Supplementary Table**

**Table S1:** Baseline characteristics of study subjects

| **Variables** | **Dapagliflozin**  **(n = 12)** | **Control**  **(n = 13)** | **P-value** |
| --- | --- | --- | --- |
| Age, years, mean ± SD | 67.0 ± 17.4 | 67.2 ± 13.4 | 0.970 |
| Male (%) | 7(58.3) | 7(53.8) | 0.821 |
| Cr, mg/dl, mean ± SD | 1.18 ± 0.32 | 1.16 ± 0.42 | 0.886 |
| eGFR, ml/min/1.73m^2^, mean ± SD | 61.3 ± 20.7 | 64.9 ± 24.4 | 0.698 |
| EF, %, mean ± SD | 42.4 ± 17.0 | 41.8 ± 16.7 | 0.924 |
| NT-proBNP, pg/ml (IQR) | 8,048.5  (3,471, 15,760) | 4,802  (3,537, 9,273) | 0.550 |
| **Underlying CV disease (%)** |  |  |  |
| Coronary artery disease | 5(41.7) | 4(30.8) | 0.881 |
| Dilated cardiomyopathy | 4(33.3) | 3(23.1) | 0.901 |
| Ischemic cardiomyopathy | 1(8.3) | 4(30.8) | 0.368 |
| Atrial fibrillation | 1(8.3) | 2(15.4) | 1.000 |
| Valvular heart disease | 3(18.8) | 2(15.4) | 0.920 |
| **Type of AHF (%)** |  |  |  |
| New onset | 5(41.7) | 9(69.2) | 0.238 |
| ADHF | 7(58.3) | 4(30.8) | 0.238 |
| **Acute respiratory failure (%)** | 0(0) | 2(15.4) | 0.497 |
| **Precipitating factors of AHF (%)** |  |  |  |
| Salt and water retention | 6(50.0) | 3(23.1) | 0.325 |
| Acute coronary syndrome | 2(16.7) | 5(38.5) | 0.443 |
| AF with RVR | 1(8.3) | 2(15.4) | 1.000 |
| Hypertensive emergency | 2(16.7) | 2(15.4) | 1.000 |
| Sepsis | 1(8.3) | 0(0) | 0.967 |
| Thyroid storm | 0(0) | 1(7.7) | 1.000 |
| **Co-morbidities (%)** |  |  |  |
| Type 2 Diabetes | 5(41.7) | 4(30.8) | 0.888 |
| Hypertension | 9(75.0) | 8(61.5) | 0.471 |
| Dyslipidemia | 6(50.0) | 7(53.8) | 0.848 |
| Cerebrovascular disease | 1(8.3) | 1(7.7) | 1.000 |
| Chronic kidney disease | 5(41.8) | 7(53.8) | 0.858 |
| Anemia | 5(41.8) | 4(30.8) | 0.881 |
| **Medications (%)** |  |  |  |
| ACEIs or ARBs or ARNi | 3(25.0) | 7(53.8) | 0.288 |
| Calcium channel blocker | 2(16.7) | 5(38.5) | 0.443 |
| Beta blocker | 6(50.0) | 5(38.5) | 0.561 |
| Furosemide | 4(33.3) | 4(30.8) | 1.000 |
| Thiazide | 0(0) | 1(7.7) | 1.000 |
| MRA | 2(16.7) | 2(15.4) | 1.000 |
| Statin | 5(41.7) | 5(38.5) | 0.870 |

ADHF, acute decompensated heart failure; AF, atrial fibrillation; AHF, acute heart failure; ACEis, angiotensin-converting enzyme inhibitors; ARBs, angiotensin receptor blockers; ARNi, angiotensin receptor – neprilysin inhibitor; Cr, serum creatinine; CV, cardiovascular; eGFR, estimated glomerular filtration rate; EF, ejection fraction; IHD, ischemic heart disease; NT-proBNP, Serum N-terminal pro B-type natriuretic peptide; MRA, mineralocorticoid receptor antagonist; RVR, rapid ventricular response.

**Supplementary References**

**S1:** Thiele K, Rau M, Hartmann NK, et al. Empagliflozin reduces markers of acute kidney injury in patients with acute decompensated heart failure. ESC Heart Fail. 2022;9(4):2233-8.

**S2:** Schanz M, Shi J, Wasser C, Alscher MD, Kimmel M. Urinary [TIMP-2] × [IGFBP7] for risk prediction of acute kidney injury in decompensated heart failure. Clin Cardiol. 2017;40(7):485-91.

**S3:** McDonagh TA, Metra M, Adamo M, et al. 2021 ESC Guidelines for the diagnosis and treatment of acute and chronic heart failure: Developed by the Task Force for the diagnosis and treatment of acute and chronic heart failure of the European Society of Cardiology (ESC). With the special contribution of the Heart Failure Association (HFA) of the ESC. Eur J Heart Fail. 2022;24(1):4-131.

**S4:** Kidney Disease: Improving Global Outcomes (KDIGO) Acute Kidney Injury Work Group. KDIGO Clinical Practice Guideline for Acute Kidney Injury. Kidney Int Suppl. 2012;2:1-138.

**S5**: Laothavorn P, Hengrussamee K, Kanjanavanit R, et al. Thai Acute Decompensated Heart Failure Registry (Thai ADHERE). Global Heart. 2010;5(3):89–95.

**S6:** Damman K, Beusekamp JC, Boorsma EM, et al. Randomized, double-blind, placebo-controlled, multicentre pilot study on the effects of empagliflozin on clinical outcomes in patients with acute decompensated heart failure (EMPA-RESPONSE-AHF). Eur J Heart Fail. 2020;22(4):713-22.

**S7:** Ngam PK, Chandra A. Positive and negative false estimate of serum creatinine. Interv Cardiol. 2017;9(4):163-7.

**S8:** Fan W, Ankawi G, Zhang J, et al. Current understanding and future directions in the application of TIMP-2 and IGFBP7 in AKI clinical practice. Clin Chem Lab Med. 2019;57(5):567-76.

**S9:** Schanz M, Shi J, Wasser C, Alscher MD, Kimmel M. Urinary [TIMP-2] × [IGFBP7] for risk prediction of acute kidney injury in decompensated heart failure. Clin Cardiol. 2017;40(7):485-91.

**S10:** Zhuo M, Paik JM, Wexler DJ, Bonventre JV, Kim SC, Patorno E. SGLT2 Inhibitors and the Risk of Acute Kidney Injury in Older Adults With Type 2 Diabetes. Am J Kidney Dis. 2022;79(6):858-67.
